# Local implementation of public health policies revealed by the COVID-19 crisis: the French case

**DOI:** 10.1186/s13012-023-01277-0

**Published:** 2023-06-23

**Authors:** Laurent Mériade, Corinne Rochette, François Cassière

**Affiliations:** IAE Clermont Auvergne, CleRMa, Research Chair “Santé Et Territoires”, University Clermont Auvergne, 11 Boulevard Charles de Gaulle, Clermont-Ferrand, 63000 France

## Abstract

**Background:**

Improving health system performance depends on the quality of health policy implementation at the local level. However, in general, the attention of researchers is mainly directed towards issues of health policy design and evaluation rather than implementation at the local level. The management of the COVID-19 crisis, especially in Europe, has particularly highlighted the complexity of implementing health policies, decided at the national or supranational level, at the local level.

**Methods:**

We conducted 23 semi-structured interviews with the main stakeholders in the management of the COVID-19 crisis in the second largest French region in order to identify the different actors and modes of coordination of the local implementation of health policies that this crisis management illustrates in a very visible way. Our methodology is complemented by a content analysis of the main guidelines and decisions related to this implementation.

**Results:**

The analysis of these data allows us to identify three levels of implementation of health policies at the local level (administrative, organizational and operational). Interviews also reveal the existence of different types of coordination specific to each of these levels of local implementation of health policies. These results then make it possible to identify important managerial avenues for promoting global coordination of these three levels of implementation.

**Conclusions:**

Although research on health services emphasizes the existence of several levels of local implementation of health policies, it offers little in the way of definition or characterization of these levels. The identification in this study of the three levels of local implementation of health policies and their specific forms of coordination contribute to a more precise characterization of this implementation in order to promote, in practice, its global coordination.

**Supplementary Information:**

The online version contains supplementary material available at 10.1186/s13012-023-01277-0.

Contributions to the literature

- In the literature, the implementation of public health policies has mainly been described on the basis of its intra-organizational determinants (mainly, readiness for implementation, organizational culture and climate, actor networks, political will).
- At the local level, the implementation of these policies is strongly influenced by the inter-organizational relationships of stakeholders and their modes of coordination, which deserve to be further documented.
- Our research shows that the local implementation of health policies takes place at three levels (administrative, organizational and operational), each characterized by specific forms of stakeholder coordination (systemic, organizational, functional, professional, normative, clinical).

## Introduction

During the COVID-19 crisis, many international studies [1–3], especially in the USA, tried to formulate very quickly a number of general recommendations to improve national health systems. The improvement of health system performance depends greatly on the quality of health policy implementation at the local level [4]. However, in general, researchers' attention is primarily focused on issues of health policy design and evaluation rather than implementation [5, 6]. Evans et al. [7] observe that the process of implementing health policies at the local level is complex because it is subject to multiple interventions that are often poorly identified.

The management of the COVID-19 crisis, specifically in Europe, has particularly highlighted the complexity of local implementation health policies decided at the national or supranational level [8]. The suddenness and unpredictability of the crisis required, at the local level, a very reactive implementation of political decisions defined by governments. This need for reactivity has highlighted, in a very visible way, the actual mechanisms of the implementation of health policies, both in their successes and their dysfunctions.

In contrast to the top-down model, which is regularly deployed in health policy, the bottom-up approach considers the implementation of health policies as a complex process which involves political actors, health organizations, medical, and care personnel working together [9]. The latter approach postulates that health policy is only fully implemented when it is reflected in the actions of the implementers and their coordination [10]. The work mobilising this approach highlights the analytical difficulties linked to the wide range of health policy implementation practices in assessing its capacity to achieve its objectives [11, 12].

To better describe these practices, a large number of intra-organizational determinants of the implementation of health policies have been described in the literature (mainly, implementation readiness, organizational culture and climate, stakeholder networks, political will) [13]. However, alongside its determinants, it still seems particularly difficult for research in health management to identify with precision the inter-organizational levels of implementation of health policies, the actors involved and the modes of coordination adopted, especially at the local level [14]. Therefore, the objective of this article is to identify more precisely the ways in which health policies are implemented locally by analyzing the coordination of actors.

In order to achieve this objective and to reflect the complexity of implementing health policies at the local level, we studied the deployment of health policies in the second largest French region during the COVID-19 crisis. France has the particularity of having a health care system that is particularly centralized in terms of decision-making. This system has very marked territorial particularities that require significant local adaptations in the way health care policies are implemented.

Our research methodology is based on a document review and analysis of the main directives and decisions for implementing health policy in the face of the COVID-19 crisis, in this region, and on 23 semi-structured interviews with a representative sample of local public decision-makers (high state's representatives in a department or region, Regional Health Agency-ARS-directors, local elected officials), health care facility managers (hospital management housing institutions for elderly dependents-EHPAD, clinics) and health care professionals (physicians, heads of department, health care managers, health care staff).

Our results identify three levels of implementation of health policies at the local level, each characterized by specific forms of coordination. The identification of these three levels allows us to begin to characterize and define the local implementation of health policies, in order to consolidate the work on the subject. This first result then allows us to identify managerial avenues for moving towards a global coordination of these three levels of implementation.

## Background

Health policy implementation is more than providing instructions around a policy document or designing a set of standard operating procedures [12]. It is a much more complex process that runs through the entire health system [7, 12]. Thus, effective implementation of health policy requires aggregating the actions of many individuals and an understanding of the ways in which these actions are or are not coordinated [15].

### Exploring gaps in policy implementation

As early as the 1970s, seminal work in political science [15, 16] identified two findings: public policies were rarely implemented as intended and the expected results were rarely achieved [6]. Pressmann and Wildavsky [16] thus coined the term “implementation gap” to describe the disconnect between goals and implementation. The longer the chain of policy implementation is, the more interrelationships there are between the links and the higher the risk of breakdown is [16].

From this time on, studies of implementation have developed strongly, using the concept of the implementation gap to compare the expectations and achievements of public policies [17, 18]. A first category of study called Deliverology (the science of achieving results) aims to describe, alongside policy design, the processes by which expected results are constructed [19]. This approach designed to measure and drive progress toward specific policy goals focuses on outcomes [19]. A second category of study called “implementation science” has sought to close the gaps between public policy goals and outcomes. This implementation science has developed tools for resolving these gaps [5] notably by mobilizing techniques developed in business administration or econometrics such as stakeholder analysis, effectiveness evaluations and mathematical modeling [20]. This second approach aims to bridge the gap between what is known to work and what can be put into practice to improve public services [6].

Studies propose quantitative or qualitative measures of intra-organizational determinants of health policy implementation at the national level [13, 21–23]. In the literature, determinants of health policy implementation [13] had been identified: implementation readiness [21], organizational culture and climate [21, 22], stakeholder relationships and networks [23], and political will to implement policy [23]. Some works mobilizing of the EPIS model (Exploration, Preparation, Implementation, Sustainment), shows that the use of bridging factors (language, contracts, structure, data exchange processes, intermediary actors, knowledge brokers) could facilitate the implementation of health policies [24–28]. These factors link the external system and the internal organisational context [25]. Like stakeholders, bridging factors can be found in all phases of diffusion or implementation [26, 28]. On the other hand, while these determinants and factors are illustrated, the levels and modes of coordination of actors for the implementation of health policies need to be more described, even though they are essential to the success of local implementation of these policies [7]. Indeed, the analysis of the levels and modes of coordination of actors in the local implementation of health policies [8] could enrich our knowledge on determinants and bridging factors.

### The issue of coordination of actors

Responsibility for implementing health policy typically falls to different actors than those who designed the policy [13]. This difference between the designers and implementers of health policies does not fail to create significant dissimilarities between the initial objectives of the policy defined, its understanding by the actors and its actual implementation [24]. These nuances often lead to delays, renegotiation of resources and responsibilities, and adjustments at all levels of the policy implementation chain during implementation [5].

The launch of a new health policy should be built by coordinating the efforts and contributions of the multiple public and private actors involved [29]. To do this, the literature often recommends that designers and implementers should be able to consider and anticipate the implementation challenges associated with particular national and local contexts [4]. This means that they should be able to recognize the characteristics of the context and anticipate the complexities of operationalizing it [9, 17]. In practice, this anticipation of problems and implementation difficulties remains difficult to construct [18].

Many public health policies are ultimately more often implemented at the initiative of administrations, organizations, or individuals with little coordination, and in the best cases, with local actors [12, 30]. Decisions to implement health policies at the local level are often made by the executive branch but are largely influenced by the needs of local stakeholders [27]. Despite this, he limited role of local actors in decision-making remains a source of tension. In these circumstances, implementation is rarely straightforward-especially as it is incrementally developed by actors whose initiatives and resources evolve in unpredictable ways [31].

In contrast to the top-down model regularly applied to health policy, many theoretical approaches view health policy implementation as a complex process that involves political actors, health organizations, and medical and health care personnel working together [9]. The bottom-up approach favoured in this work considers that policy is only fully implemented when it is reflected in the actions of those who implement it. Work mobilizing this approach highlights the analytical difficulties associated with the wide range of health policy implementation practices in assessing its ability to achieve goals [11, 12]. To better understand these implementation practices, Schnake-Mahl et al. [32] identify several levels of public health policy governance in the USA (city, county, district, state, federation of states). For their part, Purtle et al. [33] propose a taxonomy of local health policy implementation strategies in the US based on the Expert Recommendation for Implementing Change (ERIC) model, which compiles 73 implementation strategies [34]. From this compilation, these authors identify five main strategies for implementing mental health and substance abuse policies: ongoing consultation with experts, technical assistance to implementers, coalition building among implementers, development of educational materials, and use of working groups. Crable et al. [27] confirm the importance of the stakeholder technical assistance strategy during the implementation phase of substance abuse health policies in the USA. The latter study emphasizes the diversity of stakeholders’ perspectives in the implementation of health policies but also the specificity of the implementation contexts.

The identification of these strategies and stakeholders makes it possible to describe in great detail the actors involved in the implementation of health policies and their respective roles in it. The identification of these actors and their strategies can be judiciously complemented by a deepening of our knowledge of their level of intervention in the implementation of local health policies and of the modes of coordination of these actors.

## Methods

Our study aims to identify how the implementation of the health policy to fight against the COVID-19 virus was carried out locally, in particular by re-examining the relationships between the stakeholders in charge of this implementation.

In France, the territorialisation of health is not a new issue. It was first addressed in the early 1970s with the creation of the health map, which was intended to distribute healthcare provision according to territorial needs [35]. Twenty years later, in 1991, the Regional Health Organization Scheme (RHOS) was created to involve the regions in the repartition of health care supply in their territories [36]. However, this RHOS zoning was rarely reviewed over time, and it lost its effectiveness [36].

Starting in 2009, with the “Hospital, Patients, Health, Territories” (HPHT^1^) law, the State retook control of territorial health management by deconcentrating its power in health matters into the regions [29]. This deconcentration was built around the creation of the Regional Health Agency (ARS) who are responsible, by the French State, for the implementation of the health policy in their region. Thus, the State transferred to the ARSs the coordination and the piloting of all health policies from prevention to the medico-social field, including the supply of city care and hospital care [37]. However, this role assigned to the ARS was imagined for stable and non-turbulent periods [37]. The management of the COVID-19 crisis has shaken this organization.

Our case study focuses on the Auvergne Rhône-Alpes region. It is the 2nd largest region in France and chronologically the 1st region affected by the health crisis with the cluster located at Sillingy (in Haute-Savoie) which, on 7 February 2020, marked the beginning of the health crisis in France.

To carry out this study, we contacted 27 individuals in this region who were representative of the various stakeholders and we presented the purpose of the research. We identified the categories of people to interview by listing the categories of stakeholders that were mentioned in press articles, the Ministry of Health's press bulletins, freely available ministerial documents, as well as in informal discussions with elected officials in charge of local authority management and with hospital or health service administrators. 23 people agreed to undergo a semi-structured interview over the period of July 2021 to January 2022 (Table 1). The interview grid included four main themes: presentation of the interviewee and his/her background (1), consequences of the health crisis on his/her activities (measures and actions, management and changes in practices, evolution of links with other stakeholders) (2), articulation and coordination with other stakeholders and implementation of national measures as well as the place given to the local level (tools, dialogue, arrangements) (3), lessons learned from the health crisis in terms of practices and for the future (4). The interviews were conducted face to face whenever possible. Given the very tight schedule of the interviewees during this period and the geographical distance, part of the interviews was conducted by videoconference via the Microsoft Teams software without inducing any difficulty [38]. No technical problems were faced when conducting the interviews. The richness of the collected speeches confirm the first conclusions of researches on the interest of this methodology [39–41]. After obtaining formal consent from the interviewees the interviews were recorded. The 1021 min of interviews were fully transcribed and anonymized. A detailed content analysis based on manual coding of the discursive data was carried out following the recommendations of Saldaña [42]. We carried out an initial coding based on the framework of implementation science by identifying the main categories of codes from a floating reading: actors (institutional, health, status, scope of intervention, etc.), links (administrative, organisational, professional), resources (human, information, material, expertise), objectives (implementation of ministerial guidelines, defence of ethical values, etc.), and instruments (information system, steering system, space for consultation, exchange, decision).

**Table 1:** Study sample

In	Categories	Interviewee (function)	Duration	Date of interview
1	Medical territorial organisation	Hospital practitioner President of the Economic, Social and Environmental Council	1 h 07 min	July 2021
2	Health care facilities	EHPAD Director	1 h 14 min	October 2021
3	Nursing staff	Coordinating nurse	1 h	October 2021
4	Doctor	EHPAD Coordinating and occupational physician	53 min	November 2021
5	ARS Regional Strategy and Pathways Department	Regional Director of Health Agency	45 min	November 2021
6	ARS Departmental Delegation	Departmental Director	1 h 20 min	November 2021
7	ARS Departmental Delegation	Departmental Director	22 min	December 2021
8	Elected official local authorities	Vice-president of the Departmental Council in charge of territorial health	1 h	November 2021
9	Hospital management Direction of care Regional hospital	Director of Care	1 h	October 2021
10	Management of a medical establishment for follow-up care and rehabilitation	Hospital Director	51 min	November 2021
11	Hospital Centre Direction	Hospital Director	29 min	December 2021
12	Public health and University Medicine Department	Director of Public Health Department—CHU^a^	1 h 10 min	December 2021
13	Service of the State	Representative of the State	1 h 10 min	January 2022
14	Medical and territorial health care organisation	Doctor and President of the Territorial Professional Health Community	36 min	December 2021
15	Hospital management (regional structure)	Vice Director	32 min	December 2021
16	Head of Department University Hospital Centre	CHU Doctor	36 min	December 2021
17	Doctor (University Hospital Centre- CHU)	CHU Doctor	28 min	December 2021
18	CHU nursing staff	Unit Senior Manager	46 min	December 2021
19	CHU nursing staff	Service Senior Manager	50 min	November 2021
20	CHU nursing staff	Service Manager	52 min	November 2021
21	CHU nursing staff	“Covid-19” Nurse	58 min	December 2021
22	National Health Insurance Fund (CNAM)^b^	Information System Programme Director	36 min	January 2022
23	Departmental fire and rescue service	Fireman	26 min	January 2022

^a^University Hospital Center, in French, *Centre Hospitalier Universitaire (CHU)*

^b^In French *Caisse Nationale d’Assurance Maladie*

In a second step, we refined the coding around the operational, organisational and administrative levels (see Additional file 1: Appendix 1 for the final coding grid). The interviewees also provided us with documents to complement their comments, which we analyzed: white plan for hospitals, blue plan for retirement homes for the elderly, reports from the social affairs inspection, etc. In France, the white plan is a specific health emergency plan established by the 2004 law that can be implemented in public and private health establishments. It contains organizational measures intended to deal with an exceptional health situation or increased activity in a hospital. The blue plan has a similar goal for medico-social establishments for the elderly. It is drafted under the responsibility of the director of these establishments. It details the organizational procedures to be implemented in the event of a health or weather crisis.

## Results

The need to face the COVID-19 pandemic required a rapid implementation of health policy at the local level. The results of our study show several local levels of health policy implementation.

### A three-level local implementation of health policies

Our results show that the local implementation of COVID-19 crisis management policies was mainly carried out at three levels (administrative, organizational, and operational) in very different ways at each of these three levels (Table 2).

**Table 2:** The three levels of local implementation of health policies: the French case

Feature of each level	Administrative implementation	Organizational implementation	Operational implementation
Actors	- ARS^a^ - Representative of the State - Local elected officials	- GHT^b^ - CHU^c^ - Hospitals - EHPAD^d^	- Heads of department - Health care managers - Health care staff (physicians, nurses, care assistants, maintenance staff)
Implementation designers	Ministry of Health	-GHT -CHU	Department heads Managers
Implementation drivers	ARS	GHT	Managers and Health care staff
Steering	Vertical	Vertical	Horizontal
Forms of coordination	- Systemic	- Organizational - Functional	- Professional - Normative - Clinical

^a^Regional health agency (ARS) are public administrative establishments of the French State in charge of the implementation of health policy in its region. Their aim is to ensure a unified management of health in the region, to better respond to the needs of the population and to increase the efficiency of the health system

^b^Territorial hospital groupings (GHT) are a contractual arrangement, mandatory since July 2016, between public health establishments in the same geographical area, by which they undertake to coordinate around a common and graduated patient care strategy, formalised in a shared medical project

^c^University Hospital Centers (CHU) are public health establishments, which have signed an agreement with a university, or possibly with several universities. They have a triple mission of care, teaching and research. but also prevention, health education and the fight against social exclusion

^d^EHPAD are Housing institutions for elderly dependents. They are the most widespread type of French Residential care for senior citizens

These three levels of implementation can take either vertical or horizontal forms. Vertical implementation in health policy implementation is related to centralized decision-making and top-down implementation of health policies addressed to different (vertical) sectors of specialization [43, 44]. In contrast, horizontal implementation is about improving the overall health of individuals and populations (i.e., a holistic view) through intersectoral and peer-to-peer collaboration for integrated decision making and implementation [45, 46].

At the administrative level, government decisions were implemented locally in a fairly vertical manner by the ARS, based on intensified coordination with the prefectures and, to a lesser degree, with local elected officials. “The quality of communication was not always good in the field, where actors were inundated with directives and circulars that did not concern them. The information was not always targeted by the authorities. In addition, many email addresses, including some ministerial ones and those of the ARS, were inoperative because they were not updated, so requests and questions were formulated that were not processed” (I10–see Additional file 1: Appendix 2).

For this administrative level, the implementation of policy decisions at the local health system level requires continuity of these decisions with actions at the organizational and operational levels. In the case of the study area, this continuity appears relatively weak (Table 2). The coordination between actors was mainly systemic because it was carried out between the actors of this administrative level of implementation (ARS, Representative of the State, Local elected officials) without taking into consideration the other levels of implementation. Consequently, at this administrative level of implementation, the actors mainly define the general rules of crisis management and the modalities of alignment of the different health policies without really considering the contingencies of local health actors. The reason is the weakness of communication between the actors, despite an important exchange of information, and a reciprocal lack of knowledge of the contingencies of each of the actors and of their perimeters of action. Moreover, the absence of representation of health professionals from the town (general practitioners, pharmacists, medical laboratories, psychologists, etc.) did not allow them to be fully integrated into the crisis management, at least initially.

At the organizational level, based on administrative instructions, the local implementation of crisis management was carried out vertically by the GHTs, in the direction of the various health establishments in the region. “*We received quite a few recommendations from the ARS by e-mail but it was disturbing because they were in contradiction with what was happening in the services, the responses on the ground were not coordinated*” (I19).

“At first it was panic, we had contradictory orders, the doctors were asking us to come to the services but the care was deprogrammed and we had no patients, and the management was telling us to stay at home waiting for the news” (I1).

At this organizational level, local continuity of health policies also appears incomplete (Table 2). While the implementation of policies by the GHTs has made it possible to coordinate the actions of the main hospital establishments in the region, it has also excluded major local actors (private clinics, general practitioners, pharmacists) from decisions. Coordination between stakeholders was vertical and mainly organizational (coordination between the main establishments in the region) and functional (sharing of information between establishments).

Finally, at the operational level, health professionals have implemented, in a more horizontal manner, the guidelines defined by the GHTs and their establishments on the basis of strengthened coordination between the services and staff of the health establishments (Table 2).

“Although the crisis was initially managed by the ARS, it very quickly evolved, by necessity, into a form of management shared with the representative of the State, and then even more collectively, by involving elected representatives and relying heavily on the GHTs. This crisis has made it possible to establish balanced relations between the prefectures and the ARS” (I6).

This initial analysis thus enabled us to highlight, in a second phase, different forms of coordination in relation to each category of implementation (administrative, organizational and operational) (Table 2).

### Several forms of coordination in the local implementation of health policies

First, at the administrative level, the coordination of policy decisions of the local health system implies a continuity of these decisions with actions at the organizational and operational levels. At this administrative level, our results show that coordination is mainly vertical and systemic (alignment of different policies and crisis management rules). Thus, the intervention of representatives of the State and local elected officials, alongside the ARS, facilitated above all the communication and dissemination of crisis management policy guidelines. “*The crisis has allowed us to strengthen the links with the prefecture's services. We help each other, we are united in a common management that is increasingly close. We can now also work with the elected officials of the communities via the prefecture with direct relations. This allows the elected officials to know our work and recognize it*” (I7).

At the organizational level, although the vertical implementation of policies by the GHTs has made it possible to coordinate the actions of the main hospital establishments in the region, it has also excluded major local players (private clinics, general practitioners, pharmacists) from decision-making. The coordination was both organizational (coordination between the main establishments in the region) and functional (sharing of information between establishments). Thus, the GHTs have largely developed their role as coordinators of the actions of health establishments, which they had not been able to achieve since their creation in 2016. “*They have done a great deal of work in organizing the sector and mobilizing the private establishments entrusted to them” (I5). “The GHT has played a central role, the CHU has provided us with resources, it has sent us the SMUR (Mobile Emergency and Resuscitation Service)*” (I11).

At the operational level, to compensate the weaknesses of administrative and organizational coordination, the medical staff and health professionals strongly coordinated their actions (professional coordination), often through transversal coordination. To do this, they have intensified tacit and non-prescribed coordination (professional coordination) based on a broad sharing of medical information and common values (normative coordination) in order to ensure the coordination and continuity of patient care (clinical coordination). Thus, many initiatives have emerged, for example, in the relationship between health and social care in terms of support for EHPADs, or in the link between hospitals and cities through the relationships developed with CPTSs (Territorial Professional Health Communities–*Communautés Professionnelles Territoriales de Santé,* in french), which bring together professionals from the same territory to organize around a health project in order to respond to common problems. [47]. “*Today, we better understand the role of the CPTS. They are an important and concrete lever for local territorial health action" (I7). "Doctors have centralized communication because establishment management did not know what the needs were*” (I12).

## Discussion

### Towards global coordination of local implementation of health policies

The management of the COVID-19 crisis highlighted several levels of local implementation of health policies that must be considered in the definition and construction of these policies. The COVID-19 crisis also revealed the existence of different types of coordination (systemic, organizational, functional, professional, normative, and clinical) specific to each of these levels of local implementation of health policies. These different forms of coordination characterize each of these levels of implementation and constitute the main levers to be activated at each level to strengthen this implementation.

Knowledge of these forms of coordination of health actors in the territories is essential because it also reveals all the dysfunctions identified and expressed by the actors. It thus shows that the global coordination of the three levels of implementation of health policies (administrative, organizational, operational) requires the strengthening of these specific forms of coordination, but also global coordination between these three levels. In the case of the region studied, this global coordination appears relatively incomplete. The explanation given is the weakness of communication between these three levels despite a significant exchange of information and a mutual lack of knowledge of the contingencies of each and the perimeters of action of these decision-making levels. Moreover, the absence of representation of health professionals from the community (general practitioners, pharmacists, medical laboratories, psychologists, etc.) did not allow them to be fully integrated into the crisis management process, at least at first. This exclusion is a major limitation to crisis management and local implementation of health policies in France, which can be explained in part by the existence of the three levels of local implementation described in this study. Indeed, these three levels and their modes of coordination remain rather fixed and may not appear very resilient in the face of crisis situations, as they are very much centred on decision-makers and public hospitals and are not very much oriented towards community health professionals, who nevertheless represent 47% of French health professionals [46]. The COVID-19 crisis revealed the need to progressively develop new global coordination of local policy implementation, some of whose managerial levers appeared during this crisis.

Among these levers, we can mention the need to decompartmentalize these organizations by creating spaces for exchanges in order to better understand the scope of each one's action and the constraints they face, as well as to consider a real sharing and structuring of data. In addition, there is a need to integrate territorial particularities by relying in particular on the knowledge that elected officials have of their territory, because to date local authorities have not been sufficiently involved in the implementation of health policies. In order to do this, there is a real need to integrate public and private, medical and medico-social sectors, which are still very distinct and relatively distant.

In addition, the identification of these three levels of implementation and their modes of coordination should allow the implementation designers and drivers of these three levels to better determine which bridging factors [24–28] are appropriate to mobilize at each of these three levels of implementation. For example, with the emergence of the COVID-19 virus, the World Health Organization has developed a guide for the implementation and adaptation of health and social measures in the context of COVID-19 [48]. The McMaster University Health Forum has created the COVID-19 Evidence Network to support Decision-making [49] to help health care decision-makers and practitioners find and use the best evidence for responding to the COVID-19 pandemic. The dissemination and use of these guides and documents involve consultation with local communities before changes are made [48]. They are also a vehicle for integrating the three levels of health policy implementation, considering their coordination modes identified in this study.

### Contributions

The COVID-19 crisis, because of its intensity, has made it easier to identify how health policies are implemented at the local level. This allows us to contribute in three ways to a better understanding of this implementation.

First, on the theoretical level, while research on health services emphasizes the probably existence of several levels of local implementation of health policies [7, 13], it offers little in the way of definition or characterization of these levels. Our research shows that, at the local level, the implementation of health policies takes place at three levels (administrative, organizational, and operational).

Second, still on the theoretical level, for each of these three levels of local implementation of health policies, our analysis identifies several specific forms of coordination (systemic, organizational, functional, professional, normative, clinical) that allow us to begin to define each of these levels with precision.

Third, in terms of managerial implications, following the identification of these three levels of implementation and their different forms of coordination, our results suggest several recommendations to strengthen the global coordination of local health policy implementation.

### Limitations and future research

Even if the French case of local implementation of health policies is, by its complexity, rich and extensive, these contributions cannot claim to be totally exhaustive insofar as they are limited to the study of a specific region during a specific period (the COVID-19 crisis). However, given the organization and reactivity required to respond to this crisis, it can be suggested that the identification of these three levels of local implementation of health policies and their forms of coordination represents a first solid basis for a better understanding of this implementation, which seems essential to the effectiveness of public health policies. Future research, in other national contexts, will be able to mobilize this initial description of the levels of local implementation of health policies in order to validate and strengthen it.

## Conclusion

Local implementation of health policies is becoming increasingly critical to the effectiveness of health systems. A better understanding of this implementation is essential to better coordinate the decisions and actions of the stakeholders involved in this implementation. As a first step in this process, our study will allow researchers and policy makers to better understand this implementation and to initiate a more collective reflection on the most appropriate implementation modalities for each health system.

## Supplementary Information

**Additional file 1: Appendix 1**. Coding diagram. **Appendix 2.** Coding extraction

## Data Availability

The datasets used and/or analyzed during the current study available from the corresponding author on reasonable request.

## Footnotes

1 In French*, Hôpital, Patients, Santé et Territoires (HPST).*
