# Supplementary material for: Local implementation of public health policies revealed by the COVID-19 crisis: the French case
Source: Implement Sci. 2023 Jun 23;18:25. doi: 10.1186/s13012-023-01277-0 (PMC10288746; doi:10.1186/s13012-023-01277-0)
Supplement: Supplementary file 1 — Additional file 1: Appendix 1. Coding diagram. Appendix 2. Coding extraction [file 13012_2023_1277_MOESM1_ESM.docx]

**Additional file 1: Appendix 1. Coding diagram**

| **Administrative relations between state institutions (ministries, ARS) and public service operators (administrative institutional level)** | |
| --- | --- |
| Administrative coordination of health operators | -Status and territorial scale of stakeholders (national, regional, local) |
|  | -Nature and background of the relationships: old relationships active/inactivated relationships, new relationships, relationships to be built |
|  | -Coordination mechanisms: coordination unit, task force, steering groups |
| Crisis communication | -Information providers, instruments and media for dissemination |
|  | -Degree of harmonization of messages in terms of consistency, clarity and precision |
|  | -Targets of the information (quality of targeting), |
|  | -Frequency of release, delay in release in relation to ministerial announcements |
|  | -Informational assistance (interpretation, explanation) |
| Administrative steering | -Definition of rules, standards,  -Compilation and feedback on organizational coordination difficulties and operational actions |
| **Organizational relationships (organizational level)** | |
| Nature of relationships and organizational links | -Included/excluded stakeholders, coordinating stakeholder.s, pivot stakeholder.s |
|  | -Prescribed relationships, constructed relationships (formal or informal networks of stakeholders), |
|  | -Type of links: partnership, contractual... |
|  | -Resources (material, immaterial including expertise, agility) |
| Mutual knowledge / coordination | -Level of mutual knowledge of the stakeholders and their scope of action |
| Action goals | -Interpretation, adaptation, adjustment of guidelines |
| Horizontal coordination tools | -Shared information system or information sharing |
|  | -Consultation space, co-piloting, concerted actions, working groups, meetings... |
|  | -Resource sharing (concerted redeployment) |
| **Operational management (operational level)** | |
| Clinical and Professional Coordination | -Local adaptation of practices and tools,  -Articulation and adjustment of actions of medical and care staff within or outside the organization  -Affirmation of values |
| Management of resources | -Mutual adjustments of resources: human resources management (staff planning, staff distribution), materials management (masks, gowns, respirators, beds), management and reallocation of patient flows (logistics) |
| Management of information | -Monitoring and follow-up of the activity |

**Additional file 1: Appendix 2. Coding extraction**

| **Administrative relations between state institutions (ministries, ARS) and public service operators (administrative institutional level)** | |
| --- | --- |
| Administrative coordination of health operators | -Status and territorial scale of stakeholders (national, regional, local)  *« Since December 2020, we have been asked to integrate the elected representatives of the territories, i.e. the départements, parliamentarians and senators. The crisis of the covid has raised the question of the local level and the articulation of the different levels. It was a detonator. »* |
|  | -Nature and background of the relationships: old relationships active/inactivated relationships, new relationships, relationships to be built  *"The crisis has allowed us to strengthen the links with the prefecture's services. We help each other, we are united in a common management that is increasingly close. We can now also work with the elected officials of the communities via the prefecture with direct relations. This allows the elected officials to know our work and recognize it”.*  *"They have done a great deal of work in organizing the sector and mobilizing the private establishments entrusted to them".*  *"The Army was asked to help because we have the military camp next door, we had never worked with them before. They brought us the masks every week, they did an amazing job”.*  *“ we had to work hand in hand, whether or not the elected representatives agreed with the ministerial directives, things had to be done, this strengthened the relationship between the president of the departmental council and the prefect. "The mayors demanded things, but we had to resist in order to maintain a real strategy of action, this link with the mayors was finally quite new.”* |
|  | -Coordination mechanisms: coordination unit, task force, steering groups  *"This crisis has made it possible to establish balanced relations between the prefectures and the ARS" (I6)*  *“we had follow-up committees by videoconference several times a week to see how to implement the ministerial directives while integrating the constraints, they were then a little more open" "we had regular exchanges with the ministry”.*  *“we have developed reflexes of information exchange and organisational coordination which I hope will last”.* |
| Crisis communication | -Information providers, instruments and media for dissemination  *“many email addresses, including some ministerial ones and those of the ARS, were* ***inoperative*** *because they were not updated, so requests and questions were formulated that were not processed"( I10)* |
|  | -Degree of harmonisation of messages in terms of consistency, clarity and precision  *“We received quite a few recommendations from the ARS by e-mail but it was disturbing because they* ***were in contradiction*** *with what was happening in the services.”*  *"we, as representatives of the state, held audio-conference meetings with all the actors, in order to avoid informational anarchy"* |
|  | -Targets of the information (quality of targeting),  *"The* ***quality of communication was not always good*** *in the field, where actors were inundated with directives and circulars that did not concern them. The information was not always targeted by the authorities.”* |
|  | -Frequency of release, delay in release in relation to ministerial announcements  *"There was a delay in adapting to ministerial directives and the ARS, we did as best we could on the ground.”* |
|  | -Informational assistance (interpretation, explanation)  *"we cross-checked the data on vaccinations recorded in the department's centers with those of the health insurance on vaccination rates, but it wasn't easy because there were people who were vaccinated in nearby departments, or at their place of work, so we tried to make sense of these figures"* |
| Administrative steering | -Definition of rules, standards,  *"the number of vaccines received was calculated in proportion to the population, but some prefects asked for more because the population in their territory was older, so this modified the distribution. we had calculation rules given by the ministry to calculate the number of vaccination centers to be set up by department, but some adapted these rules to their advantage"*  *"we were putting in place the principles of alert, protect. It had to be done in a hyperlocal way and we weren't necessarily used to the sub-departmental level. In my opinion, this is how we should have thought about isolation to limit the spread to neighboring towns, but we had to apply the government and we had to adapt to the departmental situations.”*  -Compilation and feedback on organizational coordination difficulties and operational actions  *“ the responses on the ground were not coordinated”*  *"I had to relay the orientations of the strategy, which is decided at regional level and is aimed at implementing the health policy, which is a national policy, but obviously we have to adapt it to our context because the territories are not the same. We cannot pursue the same objectives and we will not necessarily have or implement the same means.”* |
| **Organisational relationships (organisational level)** | |
| Nature of relationships and organisational links | -Included/excluded stakeholders, coordinating stakeholder.s, pivot stakeholder.s  *"Although the crisis was initially managed by the ARS, it very quickly evolved, by necessity, into* ***a form of management shared with the prefect, and then even more collectively, by involving elected representatives and relying heavily on the GHTs****.”’*  *"The GHT has played a* ***central role****”* |
|  | -Prescribed relationships, constructed relationships (formal or informal networks of stakeholders),  *"we were able to work with the president of the departmental council, it was quite new and we quickly understood each other.”* |
|  | -Type of links: partnership, contractual...  “*We had no choice but to work together, even if it wasn't always easy to defend our interests*.” |
|  | -Resources (material, immaterial including expertise, agility)  *"we used the fire brigade who set up mobile vaccination units to go to the people, this was an important resource".* |
| Mutual knowledge / coordination | -Level of mutual knowledge of the stakeholders and their scope of action  *"Today, we better understand the role of the CPTS. They are an important and concrete lever for local territorial health action".* |
| Action goals | -Interpretation, adaptation, adjustment of guidelines  *"We had to put in place our own care arrangements on the site because they did not exist in this situation in our organisation" "We had to adapt the standards. Which we did, because there were a certain number of standards that I was asked to apply but which lacked common sense."* |
| Horizontal coordination tools | -Shared information system or information sharing  *"we were constantly on the alert for information in an informal way, we were looking for information from colleagues, we wanted to know where we were going"* |
|  | -Consultation space, co-piloting, concerted actions, working groups, meetings...  *"I have asked that every day we have a meeting at 10 a.m. of the carers to take stock of the day before in order to manage it as well as possible"* |
|  | -Resource sharing (concerted redeployment)  *“the CHU has provided us with resources, it has sent us the SMUR (Mobile Emergency and Resuscitation Service)"* |
| **Operational management (operational level)** | |
| Clinical and Professional Coordination | -Local adaptation of practices and tools,  -Articulation and adjustment of actions of medical and care staff within or outside the organisation  -Affirmation of values  **Failure of clinical and managerial operational coordination:** *"At first it was panic, we had contradictory orders, the doctors were asking us to come to the services but the care was deprogrammed and we had no patients, and the management was telling us to stay at home waiting for the news."*  ***“Doctors have centralized communication*** *because establishment management did not know what the needs were"*  *"We had a logistical problem to manage in order to have the right equipment in time to ensure hygiene and patient safety... and then how to manage the teams in the face of the unknown. We had to change our care practices as things evolved, very concretely, we had to adapt"* |
| Management of resources | -Mutual adjustments of resources: human resources management (staff planning, staff distribution), materials management (masks, gowns, respirators, beds), management and reallocation of patient flows (logistics)  *"we helped out other establishments that lacked masks, we used the local network"*  *"The CHU with the GHT has played an essential role for us, it has provided us with resources at times when the situations were very critical.”*  *"we knew that we could mobilise the CHU at very difficult times with back-up teams even if they also had a lot of difficulties".* |
| Management of information | -Monitoring and follow-up of the activity  *"Every day we bring up the numbers and use the models to try to understand what is going to happen in the next few days"* |
